# Eastern Australian estuaries will transition to tidal flood regimes in coming decades

**DOI:** 10.1017/cft.2024.12

**Published:** 2024-10-04

**Authors:** Ben S. Hague, Mandi C. Thran, Doerte Jakob, David A. Jones

**Affiliations:** 1 Science and Innovation Group, Bureau of Meteorology, Melbourne, VIC, Australia; 2 Community Services Group, Bureau of Meteorology, Sydney, NSW, Australia

**Keywords:** climate change, coastal flooding, estuaries, sea-level rise, tidal range

## Abstract

Tidal flooding occurs when coastal water levels exceed impact-based flood thresholds due to tides alone, under average weather conditions. Transitions to tidal flood regimes are already underway for nuisance flood severities in harbours and bays and expected for higher severities in coming decades. In the first such regional assessment, we show that the same transition to tidally forced floods can also be expected to occur in Australian estuaries with less than 0.1 m further sea-level rise. Flood thresholds that historically used to only be exceeded under the combined effects of riverine (freshwater) and coastal (salt water) influences will then occur due to high tides alone. Once this tidal flooding emerges, it is projected to become chronic within two decades. Locations most at-risk of the emergence of tidal flooding and subsequent establishment of chronic flood regimes are those just inside estuary entrances. These locations are exemplified by low freeboard, the vertical distance between a flood threshold and a typical high tide level. We use a freeboard-based analysis to estimate the sea-level rise required for impacts associated with official flood thresholds to occur due to tides alone. The resultant tide-only flood frequency estimates provide a lower bound for future flood rates.

## Graphical abstract

**Figure:**
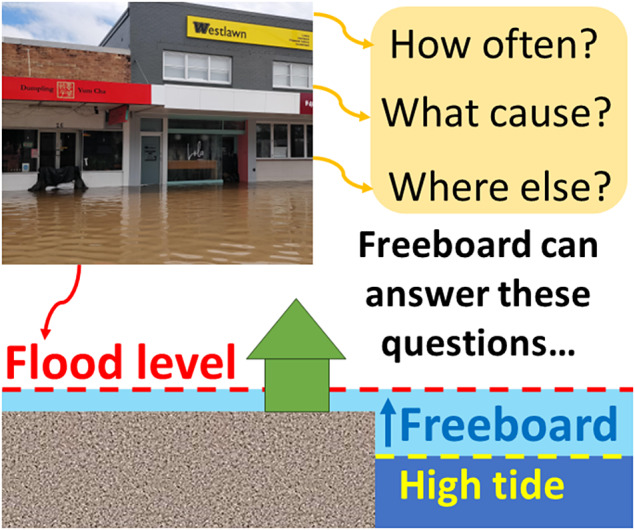

## Impact statement

This article highlights what is expected to become a key issue facing communities that live beside estuaries globally – tidal flooding. This is the first article to systematically examine the emergence of tidal flooding at severities previously only seen due to compound riverine (freshwater) and coastal (saltwater) flood events. This extends earlier work on the emergence of chronic and tidal flooding in sheltered locations such as harbours and bays where flooding occurs due to purely coastal influences. We show that once tidal flooding emerges in estuaries, flood frequencies increase rapidly with further sea-level rise. We show that the commonly used freeboard metric can easily diagnose the sea-level rise amounts that will lead to a location experiencing tidal flooding. Freeboard is the vertical distance between a flood threshold of interest and a measure of high tide. This study focuses on eastern Australia, but our findings have global implications. For example, we find that the defining feature of locations most at-risk of the emergence of tidal flooding is having lower freeboards. In Australia, these locations tend to be located just inside the mouths of estuaries, regardless of the drivers or typical magnitudes of water levels. This agrees with earlier results from the United States. In Australia, these locations often do not have official flood thresholds defined. We demonstrate an approach that can be used at any tide gauge location anywhere in the world to identify the potential for the emergence of higher-impact tidal floods using impact reports of recent flood events in conjunction with digital elevation models. Freeboard is a useful metric to identify the locations where undertaking detailed hydrodynamic modelling may need to be prioritised in a resource-constrained environment to best inform coastal adaptation policy.

## Introduction

The largest population centres exposed to coastal flood hazards are located in estuarine environments where rivers meet the sea (Temmerman et al., 2013). The most extreme floods in estuaries occur when high freshwater flows occur, sometimes coinciding with high sea levels (Moftakhari et al., 2017b; Khanam et al., 2021; Piecuch et al., 2022). Outside times of high streamflow, estuary water levels are modulated by coastal factors (Woodworth et al., 2019). Coastal water levels that used to only occur when storm surges coincided with high tides now occur under average weather conditions due to tides alone (Ray and Foster, 2016; Hague et al., 2020; Hague and Taylor, 2021; Li et al., 2022; Gao et al., 2023). However, the possibility of similar flood regime transitions occurring in estuaries has only been assessed on local scales (Helaire et al., 2020; Hague et al., 2023a; Hanslow et al., 2023; Lorenz et al., 2023).

This study aims to identify if eastern Australian estuaries are likely to experience the same transition to tide-dominated flood regimes under SLR. It also aims to understand generalisable characteristics of at-risk estuaries. For example, it is often assumed that understanding changes in flood drivers, such as the coincide of storm surge and rainfall extremes, is sufficient to quantify estuarine flood hazards (e.g., Hermans et al., 2024). Using such an approach, it has been found that locations greatly impacted in recent summer floods, such as Ballina (Bureau of Meteorology, 2022b; Dakin, 2022; Lerat et al., 2022), are generally at low risk of such floods in the current climate (Wu et al., 2018). This study aims to test the veracity of this claim leveraging information on past floods and official flood thresholds. Furthermore, we seek to understand if locations identified as low risk in the current climate will retain a lower risk than other locations as sea levels rise. We conduct the first Australian assessment of the potential for estuarine flood regime transition under SLR based on official flood thresholds.

It is physically plausible that exceedances of estuarine flood peaks previously only seen during riverine, or compound riverine-coastal, flood events may also occur due to tides alone in future. First, the effects of sea-level rise (SLR) on mean water levels in estuaries are felt well upstream (Khojasteh et al., 2023). Second, the complexities of estuarine morphology mean that tidal ranges inside estuaries can differ greatly from adjacent areas on more open coasts. In some estuaries where tidal damping occurs, tidal ranges are generally smaller in estuaries compared to non-estuarine coastal environments (Hanslow et al., 2018; Lorenz et al., 2023). In other estuaries, attenuation can occur. In both cases, these factors modulate the sensitivity of flood frequency changes to SLR (Hague et al., 2023b; Hanslow et al., 2023). Third, tidal amplitudes in estuaries will likely increase in response to SLR (Khojasteh et al., 2021, 2023) as the water near the estuary mouth will typically deepen with rising sea levels. This increases the influence of coastal-driven flooding in estuaries by reducing the distance between flood thresholds and typical heights of high tides (Ralston et al., 2019; Hague et al., 2023a; Pareja‐Roman et al., 2023).

The emergence of tidal flooding in which tides alone exceed a flood threshold, is a harbinger of the imminent emergence of chronic coastal flooding (Sweet and Park, 2014; Thompson et al., 2021). The commencement of chronic and tidal flood regimes leads to fundamental changes in flood hazards. First, tidal flood peaks and durations are highly predictable (Hague et al., 2020). Future floods can be predicted well in advance and emergency managers can take more pre-emptive than reactive approaches. Second, the annual average economic impact of cumulative nuisance flooding may exceed that of rare episodic floods which are the primary drivers of losses in the present climate (Moftakhari et al., 2017a; Ghanbari et al., 2020).

The definition of flood hazards is inherently subjective. This is acknowledged by national meteorological and oceanographic services, such as Australia’s Bureau of Meteorology. They collaboratively defined flood thresholds with local flood and emergency managers based on local assessments of the impacts of past events (Bureau of Meteorology, 2013). For example, minor flooding is typified by closures of minor roads, bicycle and pedestrian paths, and flooding of property below the floor level. These intrinsic diagnostic properties of minor flooding have been used in defining impact-based minor flood thresholds for coastal locations in Australia (Hague et al., 2019, 2022). Flooding of buildings above floor levels and evacuation are features of moderate and major floods (Bureau of Meteorology, 2013). Major flooding is most extensive and may additionally lead to utility services being impacted and town being isolated. The United States National Weather Service adopt a similarly tiered approach with advisory (“minor flooding possible”), watch (“significant impacts possible”) and warning (“threat to life and property”) (National Weather Service, 2024).

## Methods

### Observed water levels

Water levels were obtained from national gauge network locations that support flood forecast and warning services at the Bureau of Meteorology. This subset of gauges was identified as tidally influenced by inspection of the timeseries for 12 or 24-hour periodicities, and the expert judgement of an operational hydrologist. Water level data are reported with respect to the datum used for flood forecasting and risk mitigation. This usually the Australian Height Datum (AHD), approximately 1966–1968 mean sea level. However, some locations (e.g., those in the Richmond River catchment) have gauges which report to low water datums. Record lengths and other metadata are provided in Supplementary Table S1. Data are archived in a raw state, so quality assurance was applied to the data used in this study. Additional data for Ballina case study was provided by Manly Hydraulics Laboratory on behalf of the NSW Department of Planning and Environment (NSW DPE). Pre-2022 data for Ballina Breakwall was obtained from ANCHORS (Hague et al., 2021). All Ballina water levels are reported with respect to the Richmond River Valley Datum (RRVD), which is approximately the lowest astronomical tide.

### Flood thresholds

Official impact-based minor, moderate, and major flood thresholds (e.g., Bureau of Meteorology, 2013, 2022a) are used in this study.

For Ballina, we match recorded water levels to the impacts reported in the vicinity of the gauges (following Hague et al., 2022), as no official thresholds are defined. The heights of these water levels are verified based on land height estimates obtained from the airborne 2019 NSW Marine LiDAR Topo-Bathy surveys (State Government of New South Wales and Department of Planning and Environment 2019) accessed using the “Elevation at Point” tool on Elvis – Elevation and Depth – Foundation Spatial Data portal (https://elevation.fsdf.org.au/). These are based on a 1 metre resolution digital elevation model (DEM). Such data meet the Australian requirements of a fundamental vertical accuracy of 0.3 m, making them among the highest accuracy DEMs available worldwide (Wilson and Power, 2018; Kulp and Strauss, 2019).

### Quality assurance

Water level data are converted to hourly frequency by taking the top of the hour observation as representative of that hour for flood hazard assessment purposes. Secondly, we remove all values that differ by more than 0.5 m from their adjacent hourly value to remove spikes. This is performed iteratively until the values on either side of the missing data period differ by 0.5 m or less. Whilst this may result in the removal of more data than necessary, tidal analysis is assumed to remain robust with short (e.g., 1 data-year) records (e.g., as demonstrated by Pawlowicz et al., 2002). Third, any sites with less than 5 data-years and less than 70% non-missing data were removed. These are 75 suitable Australian estuarine tide gauges that have official flood thresholds defined as part of the Bureau of Meteorology flood forecasting and warning service and meet these data requirements.

This quality assurance process resulted in the removal of all but one location from outside Australia’s eastern seaboard (Barrack St, Perth). It also removed several locations with large tidal range, mostly from northern Queensland, as these can have water levels change by 0.5 m per hour due to astronomical tides. Previous research suggests that unless coastal flooding is already frequent these are the least likely locations to see chronic flooding this century (Hague et al., 2023b). No detrending was applied due to the short record available. The Perth gauge is excluded from much of the analysis due to its geographic distance.

### Harmonic tidal analysis

Harmonic analysis was performed on quality-assured hourly observations, using TideHarmonics (Stephenson, 2017). A total of 114 constituents were used with nodal corrections to produce a timeseries of hourly astronomical tide levels from 2003 to 2022 inclusive. These represent an estimate of water levels under average weather and climate conditions. The highest and lowest astronomical tides (HAT, LAT) for each gauge are the maximum and minimum values of the tidal timeseries, respectively. The annual and monthly highest astronomical tides (AHAT, MHAT) are the average of the maximum hourly tide levels recorded in each calendar year or month, respectively.

### Freeboard as a metric to estimate SLR required for flood regime transition

**Figure 1.**
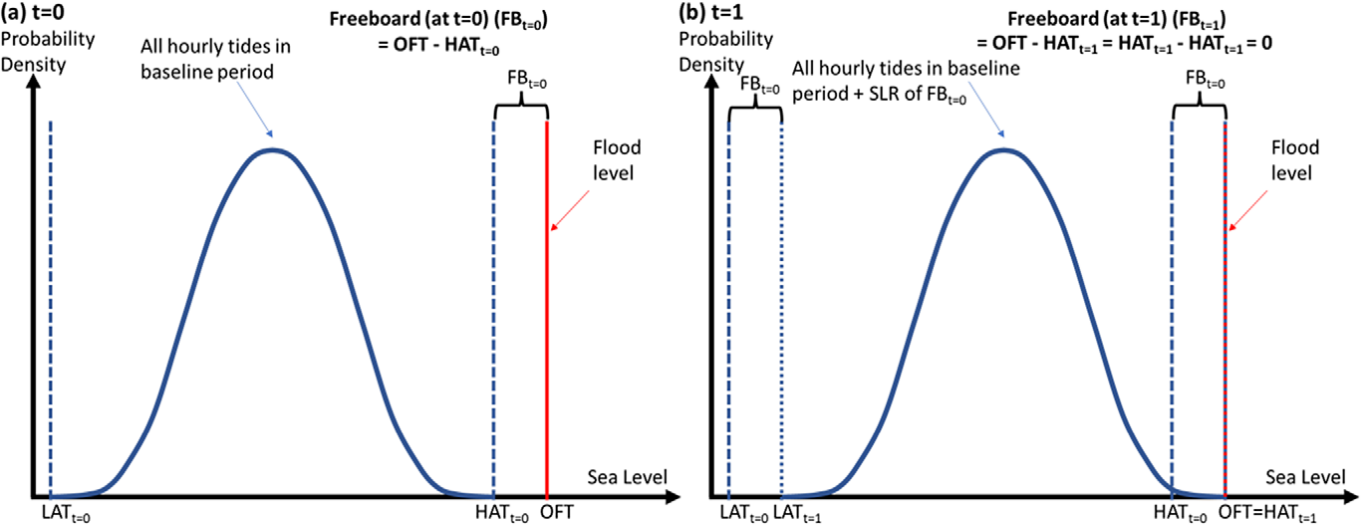
Freeboard (FB) changes under sea-level rise and stationarity assumptions. LAT and HAT are the lowest and highest astronomical tides, respectively, and OFT is the official flood level. Refer to the text for further interpretation.

Freeboard is the vertical distance between a flood threshold and some measure of high tide over a given period (Sweet and Park, 2014; Dusek et al., 2022; Ritman et al., 2022; Hague et al., 2023b; Figure 1). Here we consider freeboards based on the highest astronomical tide (HAT), annual average highest astronomical tide (AHAT), and monthly average astronomical tide (MHAT). For example, if MHAT is 0.8 m below the minor flood threshold we say the minor-MHAT freeboard is 0.8 m.

Most coastal flood hazard assessments assume stationarity of flood threshold and stationarity of water level variance (e.g., Fox-Kemper et al., 2021; Thompson et al., 2021; Sweet et al. 2018). The stationarity of flood thresholds implies that the water levels of local concern do not change through time (Figure 1). Stationarity of variance implies that future water level distributions can be obtained by increasing all water levels in a timeseries or distribution by a constant offset equal to the SLR expected at the future time (Figure 1). Hence, the differences between the two water levels are exactly equal to the SLR amount that is required for the higher level to occur as frequently as the lower level does at present (Taherkhani et al., 2020; Hermans et al., 2023). This means the increase in mean sea level (MSL) required for the emergence of tidal flooding is equal to the freeboard (Hague et al., 2023b). For example, a minor-MHAT freeboard of 0.8 m implies that 0.8 m SLR will lead to coastal-driven water levels that currently exceed MHAT to exceed the minor flood threshold. This is the basis upon which freeboard is used as a metric to estimate SLR amounts that lead to the emergence of tidal flood regimes in estuaries.

This is demonstrated in Figure 1. This shows a schematic of the equivalence between freeboard and SLR required for the flood threshold to be equal to HAT, implying the emergence of tide-only flooding. (a) Shows the situation at some start time *t* = 0, with HAT and LAT as the lowest and highest hourly tide levels in the baseline period (dashed blue lines), and freeboard (FB) as the difference between the official flood threshold (OFT, red) and HAT. (b) Shows the same situation under SLR equivalent to the freeboard at time *t* = 0 (FB_*t*=0_), with values of HAT and LAT increased by FB_*t*=0_ (dotted blue lines), hence freeboard at *t* = 1 (FB_*t*=1_) is 0, implying tide-only flooding can occur. The assumption of stationary of flood threshold can be seen by the red flood threshold line not changing in height between the two scenarios. The assumption of stationarity of variance can be seen by the new HAT and LAT values (and all water levels in hourly tide level distribution) all being increased by the same amount going from *t* = 0 to *t* = 1, equal to the SLR increment of FB_*t*=0_.

**Figure 2.**
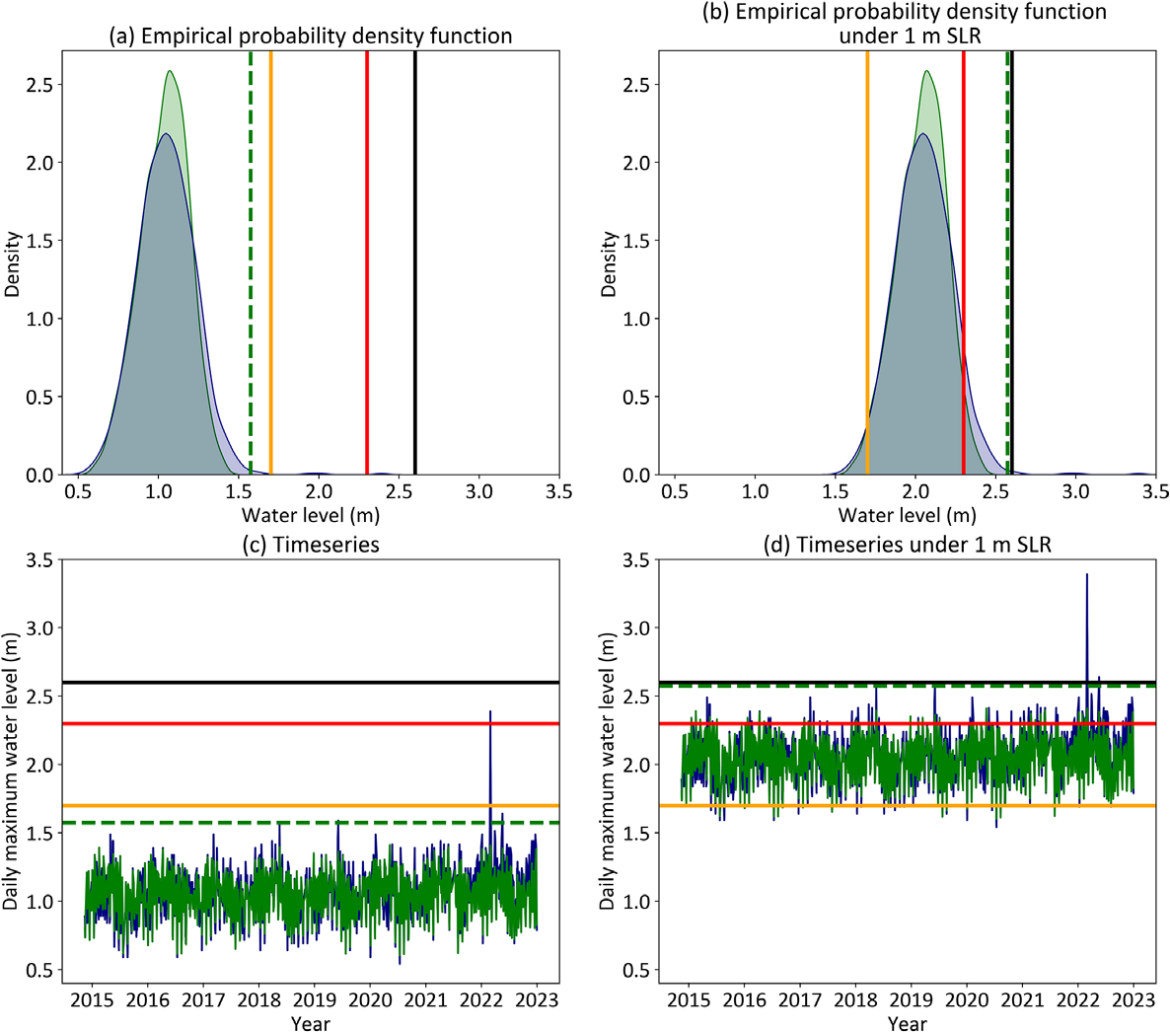
Demonstration of how flood threshold changes from being far in the (a) upper tail of the distribution, or equivalently, (b) well above typical water levels, to (c) being in the main bulk of the distribution, or equivalently (d) within the range of typical water levels. This uses the example of Hawthorne in the Brisbane River. Daily maximum water levels are plotted in all sub-figures.

This freeboard-based approach is validly applied in situations where riverine influence on water levels is negligible. This includes the present application as coastal and tidal floods occur much more frequently than extreme rainfall events that drive riverine and compound floods. The streamflow extremes that have led to past significant riverine floods are likely to remain no more frequent than annual occurrences with climate change (Slater et al., 2021; He et al., 2022). In contrast, high sea levels that presently lead to coastal flooding will become chronic occurrences in coming decades (Thompson et al., 2021; Hague et al., 2023b). Hence, tidal and frequent coastal flooding is a function of the bulk of the water level distribution, not the extreme tails where compound floods reside (Hanslow et al., 2018; Ghanbari et al., 2019; Hague et al., 2023b; Figure 2). The proportion of compound floods out of the overall number of exceedances of these thresholds will therefore be small. Similarly, projected differences between increases in compound flood peaks and coastal water levels (Moftakhari et al., 2017b; Kumbier et al., 2018; Ghanbari et al., 2021) are not expected to impact results.

This is demonstrated in Figure 2, which shows how flood thresholds change from being in the upper tail of the water level distribution (a and c) to within the bulk of the distribution under SLR (b and d). This is shown from the perspective of an empirical density function (a and b) and timeseries (c and d) of daily maximum analysed astronomical tide level (green) and observed water levels (blue). Current official minor, moderate and major flood thresholds are shown as orange, red and black lines respectively. HAT is shown as a dotted green line.

Stationarity of variance is a stronger assumption for estuaries than other coastal environments due to changes in tidal range (e.g., Hague et al., 2023a; Pareja‐Roman et al., 2023), and sedimentary processes (Khojasteh et al., 2022; Thom et al., 2023). SLR and global reductions in sediment supply may lead to deeper estuaries with larger tidal ranges in future, especially in constricted estuaries (Leuven et al., 2019; Khojasteh et al., 2023). Hanslow et al. (2023) provide a simple approach to account for the effect that increases in tidal range have on coastal flood projections that could be applied in future studies that have sufficient data to compute robust trends in tidal range.

## Results and discussion

### Baseline minor, moderate and major flood rates at Australian coastal gauges

Minor, moderate, and major floods have occurred episodically in Australian estuaries over the last two decades. Half of the locations experience minor flooding no more than once every 4.5 years on average. The top 10% minor-flood-prone locations experienced minor flooding at least every other year on average. Moderate flooding occurs once a decade on average across the 75 gauges. In other words, in an average year, 7 or 8 of the 75 gauges expect to see moderate flooding. Major flooding has occurred at 31 of 75 gauges over their observational records (Supplementary Table S1). Minor, moderate, and major flooding occurs at twice the frequency at NSW gauges than at Queensland gauges. A previous study argued that NSW has lower coastal flood hazards than other states as storm surges and extreme rainfall are largely independent there (Wu et al., 2018). This is not true based on observed flood rates. Other more impact-based approaches may be better suited for identifying spatial patterns in flood hazards across national and regional domains than metrics based solely on water levels or drivers.

In years where minor flooding occurs, it generally only occurs on a small number of days in that year. Half of all locations average no more than 4.2 days of minor flood threshold exceedance in years when flooding occurs. These floods are predominantly driven by fluvial factors, with compounding coastal influences in some circumstances, as reflected in the Bureau’s Service Level Specification for the warning services thresholds were defined for. The rareness of minor, moderate and major floods in the present climate adds further weight to our argument that the influence of riverine factors on the total number of coastal floods will be negligible once sufficient SLR occurs for these thresholds to be exceeded by tides alone (e.g., Figure 2d).

### Emergence of tide-only flooding

**Figure 3.**
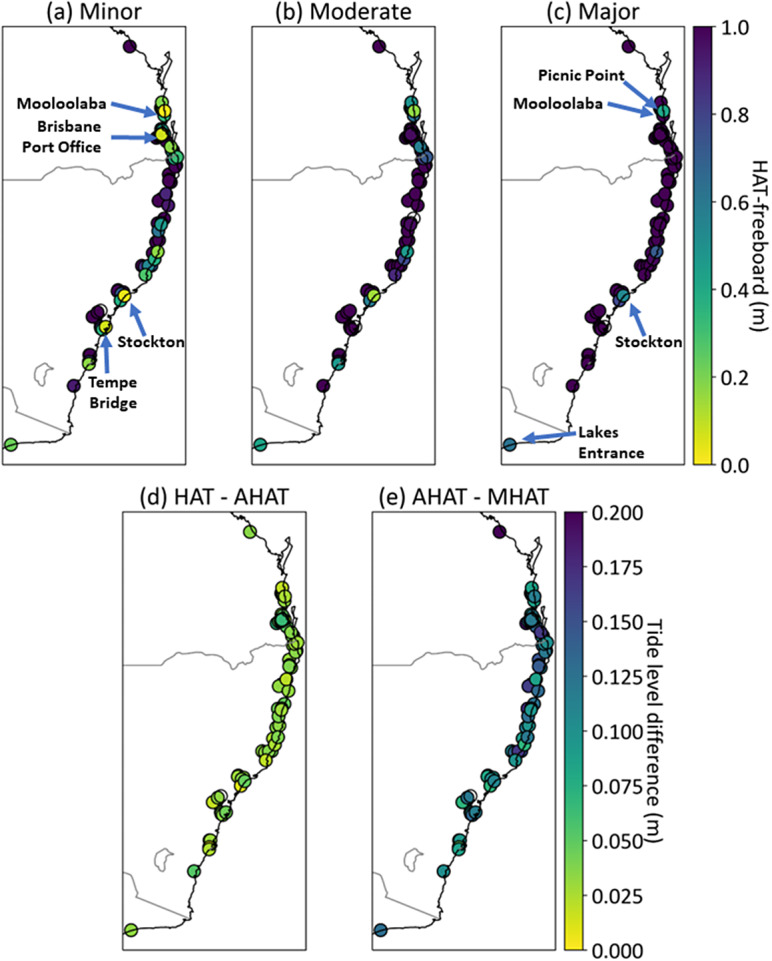
HAT-freeboards associated with (a) minor, (b) moderate, and (c) major flood thresholds. These equal the SLR for tidal flooding to emerge at each severity. Differences between (d) HAT and AHAT, and (e) AHAT and MHAT. These equal the additional SLR for tidal flooding to occur on average once per year and once per month, respectively, once tidal flooding has emerged. Results are also provided in Supplementary Table S1. Barrack St (Perth, Western Australia) is not shown.

Analysis of freeboards (Supplementary Table S1) indicates that many locations that presently only experience compound riverine-coastal flooding will experience flooding due to tides alone this century, based on current SLR planning guidelines (McInnes et al., 2015; Norman and Gurran, 2018). Minor-HAT freeboard is the SLR required for the emergence of tide-only flooding (Hague and Taylor, 2021). Thirty locations have minor-HAT freeboards of less than 0.7 m. Of these, 11 have minor-HAT freeboards of less than 0.2 m. These locations are expected to see the emergence of tide-only flooding in the coming decades (Fox-Kemper et al., 2021). Four gauges expect tide-only minor flooding with less than 0.1 m additional SLR (Figure 3a) – Mooloolaba (Sunshine Coast, QLD), Stockton (Newcastle, NSW), Tempe Bridge (Sydney, NSW) and Brisbane Port Office (QLD). The emergence of moderate and major tidal floods is also expected under 0.7 m SLR. 12 locations have moderate-HAT freeboards of less than 0.7 m (Figure 3b). Four locations expect major tide-only flooding to emerge with SLR amounts less than 0.7 m (Figure 3c) – Mooloolaba (0.42 m), Stockton (0.54 m), Picnic Point (Maroochy River, QLD) (0.61 m), and Lakes Entrance (Victoria) (0.62 m).

The 30 locations where 0.7 m SLR leads to the emergence of tide-only flooding represent 40% of all studied locations. Given not all tidal estuaries in Australia have official flood thresholds it is likely that other locations not identified here may also be expected to see the emergence of tidal flood regimes this century. Further, offsets between flood thresholds are less than 0.3 m in several locations (Supplementary Table S1). This is less than the Australian standard for DEM vertical accuracy over bare ground (Wilson and Power, 2018). Verification of DEM-derived flood thresholds may be required to ensure the resultant flood hazard assessments are reliable (e.g., Habel et al., 2020). The freeboard-based methods demonstrated in this study provide a method to identify locations where higher-accuracy local-scale modelling may be required to inform adaptation pathways (Hanslow et al., 2023). For example, digital elevation models based on LiDAR data from unmanned aerial vehicles (UAVs) can achieve vertical errors less than 0.1 m (Chen et al., 2020). These local-scale models can assess if adaptation triggers may be reached before flooding becomes chronic or tidal (Buchanan et al., 2019; Allison et al., 2023).

### Establishment of tidal and chronic flood regimes

Once tide-only flooding emerges, flood frequencies increase rapidly and predominantly tidal flood regimes become established (Sweet and Park, 2014; Thompson et al., 2021; Hague et al., 2023b). We show this is also true for Australian estuaries, as there are small differences between HAT-, AHAT- and MHAT-freeboards (Figure 3d,e and Supplementary Table S1). Across all 75 locations, none have more than 0.07 m offset between HAT and AHAT, with an average of less than 0.04 m. This means it takes only an additional 0.07 m SLR for tide-only flooding to occur annually following its first occurrence. The 12 locations that have moderate-HAT freeboards less than 0.7 m, also have moderate-AHAT freeboards less than 0.7 m. Only 0.11 m further SLR leads to tide-only flooding occurring monthly on average. Therefore, less than 0.2 m SLR is enough for tidal flooding to go from being non-existent to chronic. Following from the freeboard and SLR equivalence shown in Figure 1, DEMs that conform to the Australian standard of 0.3 m vertical accuracy likely cannot distinguish between areas with no tidal flooding and those with chronic tidal flooding. Further, 0.2 m is only two decades’ worth of SLR (under a higher SLR scenario) if tidal flooding emerges after 2050 (Fox-Kemper et al., 2021). This result applies regardless of flood severity as the difference between major-MHAT and major-HAT freeboards is the same as the difference between minor-MHAT and minor-HAT freeboards (Supplementary Table S1).

Observed exceedances of AHAT and MHAT are much higher than their expected frequency of exceedance under tides alone. For example, AHAT and MHAT are exceeded on average 28 and 61 days per year. These annual flood rates are comparable to accepted definitions of how frequently flooding needs to occur to be considered chronic (Sweet and Park, 2014; Thompson et al., 2021; Gold et al., 2023). Freeboard metrics provide robust estimates of the *maximum* possible SLR amount that will lead to the emergence of tide-only flooding of some frequency. However, it is likely that less SLR will be sufficient for the establishment of chronic flood regimes. First, storm surges lead to higher numbers of coastal floods than expected under the average weather conditions implied in harmonic tidal analysis (Hague et al., 2022, 2023b). Second, compound floods will continue to occur in addition to coastal floods due to tides alone, and coastal floods due to the combination of tides and surges. Third, increases in tidal range may have led to larger increases in HAT than expected due to mean SLR alone.

**Figure 4.**
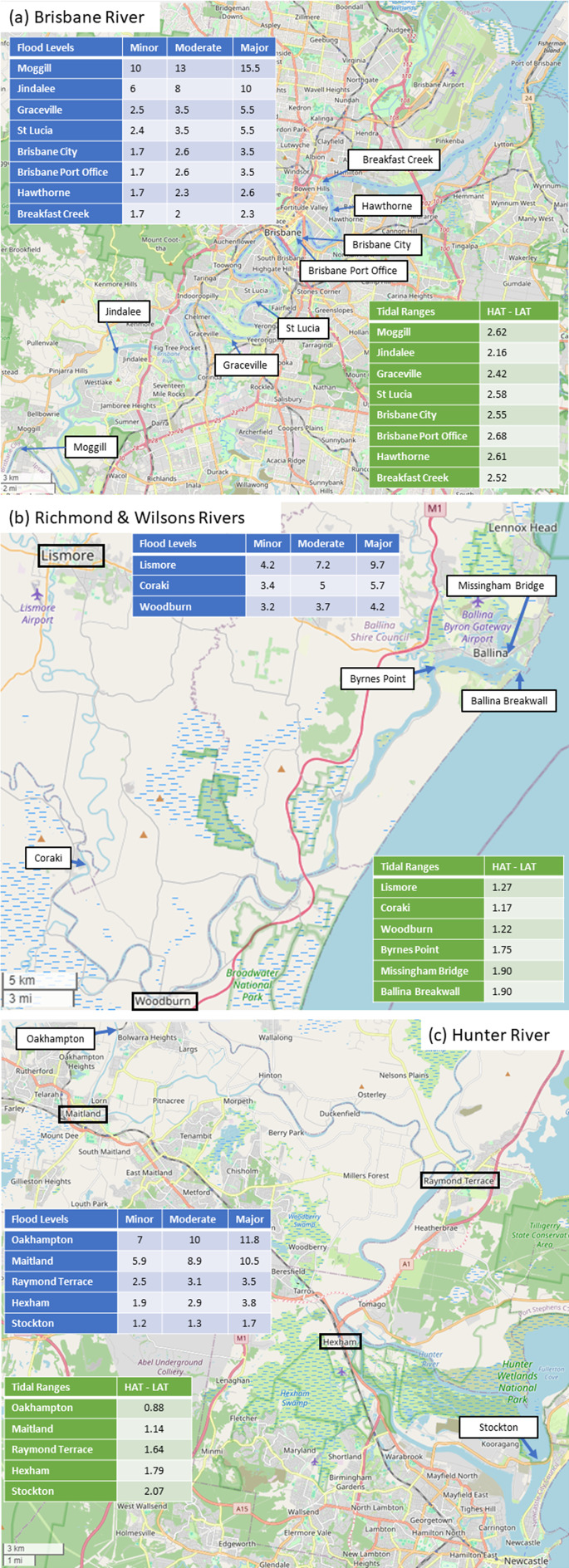
Flood thresholds decrease in height the closer to the river mouth a location is. Tidal ranges tend to increase in height the closer to the river mouth a location is. Examples are shown from (a) Brisbane River, (b) Richmond and Wilsons Rivers, and (c) Hunter River. All flood thresholds were reported in metres AHD in (a) and (c), and Richmond River Valley Datum in (b). Base map from Open Street Map.

The establishment of chronic minor flood regimes in the lowest reaches of many estuaries is inevitable without flood risk mitigation that eliminates all flood impacts associated with exceedances of the present-day minor flood threshold. Global mean sea level rises exceeding 1 m are inevitable due to past emissions (DeConto et al., 2021; Box et al., 2022). Mooloolaba, Stockton (Newcastle), Picnic Point (Maroochy River), Lakes Entrance, Settlement Point (Port Macquarie) and Belmont (Lake Macquarie) all have Major-AHAT freeboards less than 1 m (Supplementary Table S1). This means that a future state where tides alone exceed historically significant flood peaks is a matter of *when* rather than *if* for these locations. The main sources of uncertainty over the timing of emergence of frequent flooding are how rapidly SLR responds to past and future emissions, and how flood thresholds relate to high water levels (i.e., freeboard). Metrics that do not consider how high water levels must be to cause flooding, such as sea level allowances and amplification factors (Buchanan et al., 2017; Woodworth et al., 2021), are therefore unlikely to be useful for assessing the potential for flood regime transitions in estuaries. The only locations with minor-AHAT freeboards less than 1 m are those several kilometres upstream, as flood thresholds increase upstream (Figure 3a). Figure 4 shows this phenomenon applies even more to moderate and major flood thresholds.

### Identifying characteristics of locations most at-risk of flood regime transition

Locations with smaller tidal ranges and lower freeboards are most at-risk of flood regime transition. First, the 12 locations that expect moderate tidal flooding with less than 0.7 m SLR (Figure 3b) are those where water level variability is smallest. The mean HAT–LAT tidal range of these “at risk” locations is 1.4 m, compared to 1.56 m for the overall mean. Both of these numbers are less than typical coastal tidal ranges (Hague et al., 2022, 2023b), suggesting Australian estuaries may be in general at greater risk of chronic flooding than other coastal locations globally. Second, the same 12 locations *all* have minor-HAT freeboards of less than 0.4 m. This is much less than the *average* of all 75 locations, 1.34 m. This is also true for major-HAT freeboards. The average across all sites is 3.33 m, whilst the average across the 12 most at-risk sites is 0.86 m. Small tidal ranges and lower freeboards are known characteristics of locations identified as most at risk of chronic flooding (Hague et al., 2023b). Locations that experience the most frequent or severe flooding due to storm surges are not necessarily the same locations at risk of frequent future flooding. Analogously, our results suggest that whether a location experiences predominantly riverine or predominantly coastal flooding at present has little bearing on whether it will transition to experience chronic tidal flooding in the future. The locations most at risk in future are not necessarily the most at risk now. This means that assessments of present-day compound flood risk may be of limited use for assessing future compound flood risk.

Being situated immediately inside the mouth of a tidal river or lake may be another good indicator for locations’ potential for highest future compound flood risk. Heights of minor, moderate and major flood thresholds decrease with decreasing distance a gauge is from the estuary mouth in the Brisbane River (Figure 4a), Richmond and Wilsons Rivers (Figure 4b), and Hunter River (Figure 4c). This is also true in other rivers and estuaries where two or more gauges are installed (Supplementary Table S1). Concerningly, the locations most immediately inside the estuary mouth generally do not have official flood thresholds defined. This is also evident in Figure 4 – Newcastle, Ballina, and Brisbane’s airport, port area, and surrounding suburbs do not have defined flood thresholds. These are locations that could be the most at risk but could not be included in this study’s assessment. The lack of flood thresholds in these locations is due to flood warning services being targeted towards fluvial floods (Bureau of Meteorology, 2013), which have historically led to the largest impacts and losses (Callaghan and Power, 2014). These impacts have generally occurred further upstream where flood peaks (and hence, flood thresholds) are higher. We suggest that identifying large population centres just inside estuaries may be a useful first step for identifying possible gaps in water level datasets and impact databases. An example of how this can be done is shown for Ballina in the next section.

Previous studies have identified present-day flood hotspots as locations where large storm surges and large riverine discharges coincide temporally (Wu et al., 2018; Couasnon et al., 2020; Nasr et al., 2021). Several studies have argued that future changes in storm surges and riverine discharges will be the most important factors determining future flood hazards in these areas (Zscheischler et al., 2018; Bevacqua et al., 2020). Our results suggest otherwise. First, the emergence of tide-only flood cannot be captured if only considering storm surges and riverine discharges without astronomical tides (Ray and Foster, 2016; Hague and Taylor, 2021). Second, estimating the emergence of tide-only flooding, or indeed any changes in flood frequency, depends on knowing the flood threshold (Rasmussen et al., 2022; Hague et al., 2023b; Sun et al., 2023). The large variations in flood thresholds within the same river system (Figure 4) suggest that more factors than simply storm surge and riverine flow peak values determine flood thresholds. For example, the existence of assets that could be inundated during high water level events is an important factor in determining whether a flood hazard of some severity exists. Third, the locations which are most likely to experience future frequent flooding in Australia are not the same locations where large storm surges and large riverine discharges coincide. It could be expected that this is also true globally, given that small tidal range and lower freeboards are reliable global indicators for emerging chronic flood hazards (Hague et al., 2023b).

### Assessing the potential for flood regime change without official flood thresholds: Ballina, NSW

**Figure 5.**
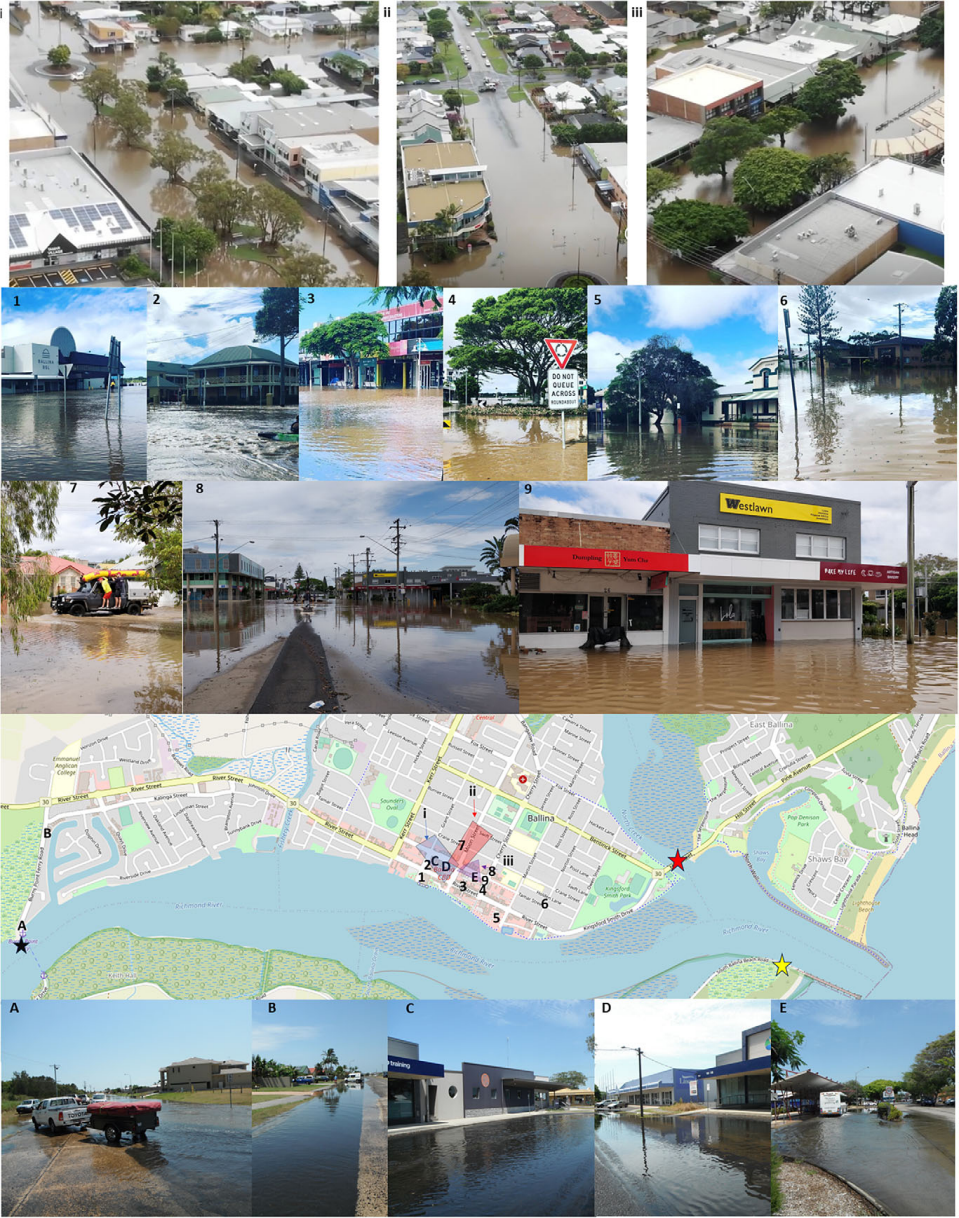
Images of flood impacts in Ballina with the location (or extents if aerial photograph) depicted in the image shown on the map. Upper images labelled with numbers from the 1–4 March 2022 compound flood event, reproduced with permission of local photographers Hover Images (i–iii), Aimee Keenan (1–6) and Frank Coughlan (7–9). Lower images labelled with letters are from 2 to 3 January 2014 king tide event, reproduced from Witness King Tides flickr, under CC-BY-2.0. Credit Tom Coster (A) and Garry Owers (B–E). Water level gauge locations are shown as stars – Byrnes Point (black), Missingham Bridge (red) and Ballina Breakwall (yellow).

The previous section showed that locations with large population centres just inside estuaries are likely most at risk of flood regime change with SLR. Ballina is a town of 46,000 people located at the mouth of the Richmond River (Figure 4b). It has no official flood thresholds defined, but experiences frequent nuisance tide-driven flooding due to high tides and storm surges (Hague et al., 2022). One such coastal flood event occurring during king tides in January 2014 (Figure 5). Ballina experiences much more severe flooding during periods of high streamflow in the Richmond River, especially when these coincide with high tides. One such compound flood event occurred over 1–4 March 2022 (Bureau of Meteorology, 2022b; Dakin, 2022; Lerat et al., 2022; Figure 5). Impacts during this flood included loss of communications and electricity, damage to thousands of homes and businesses despite precautionary sandbagging, hundreds of emergency rescues despite an evacuation order, and relocation of patients to a makeshift hospital on higher ground (Ciccarelli et al., 2023; Wahlquist and Tondorf, 2023). We use the March 2022 and January 2014 events to demonstrate that freeboard metrics can be applied robustly to “event thresholds” (i.e., the highest level recorded over a multi-day flood event), to assess the potential for flood regime changes in locations without official flood thresholds.

**Figure 6.**
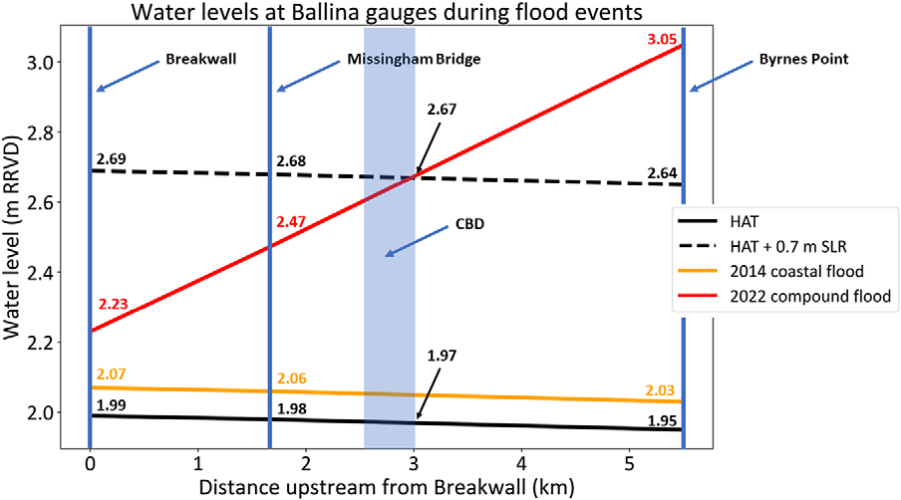
Water levels (in m) along the lower Richmond River based on observations at Breakwall, Missingham Bridge and Byrnes Point. For locations refer stars in **Figure 5**. Observations from 2014 coastal flood are shown in orange and 2022 compound flood is shown in red with corresponding values reported in m RRVD. Estimates of HAT from harmonic tidal analysis are shown in black, with estimate of HAT under 0.7 m SLR dotted. The approximate location of Ballina CBD is shown as shaded blue area, with estimate of flood peak indicated by number with black arrow, as described in the text.

Estimating freeboards requires estimates of HAT and a flood threshold. Flood thresholds for Ballina are estimated by identifying water levels at gauge where water levels are representative of reported impacts at the time impacts are reported (Hague et al., 2019, 2023a). Estimating freeboard for the 2022 compound flood event was complicated by a strong hydraulic gradient throughout the lower Richmond River and no gauges in the immediate vicinity of the CBD where most impacts were reported (Figures 5 and 6). The Breakwall recorded 2.23 m, whilst just over 1.5 km upstream Missingham Bridge recorded 2.47 m. Byrnes Point, in West Ballina 5.5 km upstream from the Breakwall, recorded 3.05 m. (Note, these water levels are relative to a low-water datum, rather than AHD). Assuming a linear hydraulic gradient along the length of estuary is justified based on the observed linear relationship between flood peaks of each of the gauges (Figure 5). Applying this to estimate water levels at the western edge of the CBD suggests a flood threshold of 2.67 m is an appropriately conservative estimate for the water level that led to the impacts in Figure 5. Similarly, a HAT of 1.97 m can be estimated. This yields an event freeboard of 0.7 m.

Several other lines of evidence also suggest that 2.65 m is an appropriate estimate for the 2022 flood threshold in the Ballina CBD. First, based on apparent flood depths in the photographs, the water levels in 2014 coastally forced event and the 2022 compound-forced event depicted in Figure 5 (images i–iii and 1–9) are unlikely to differ by more than 0.6 m. The 2014 event recorded water levels between 2.03 m and 2.07 m along the estuary (Figure 6). For example, higher parts of footpaths remain visible in image i, despite the road also experiencing flooding during the 2014 event (images C and D in Figure 5). Second, DEM data indicates heights of between 1.3 m and 1.8 m AHD in the region where flooding is pictured in Figure 5. For example, the bus bay (location E, curved roof in iii) is at 1.3 m elevation but the intersection of Crane and Moon St (the non-flooded intersection in the centre of ii) is at 1.8 m. 1.8 m AHD is equivalent to 2.64 m RRVD (Modra, 2013).

Estimating freeboard for the coastal-driven 2014 flood is more straightforward as water levels at all three gauges (Breakwall, Missingham Bridge, Byrnes Point – see Figure 4b) in the Ballina area recorded similar values (Figure 6). Estimates of event freeboards were identical for all gauges, at 0.08 m as HAT similarly varies between the three gauges (Figure 6). Hence the event freeboard for CBD impacts can be assumed to be 0.08 m. The constant freeboard along the Richmond River during this event (Figure 6) suggests that it is appropriate to assume that SLR will lead to all three gauges experiencing the same increases in water levels for future *coastal* driven flood events (Hanslow et al., 2018; Fox-Kemper et al., 2021). HAT has been exceeded on average 4.2 days per year by storm surges at the Breakwall over the 20-year epoch 2003–2022. It follows from the difference between 2.67 m and 1.97 m being 0.7 m that 4.2 days per year is the expected frequency of March 2022 flood impacts under 0.70 m SLR (Taherkhani et al., 2020; Hague et al., 2023b). This 0.7 m is also an estimate of the SLR required for tides alone to be sufficient to lead to the impacts experienced in March 2022. Whilst tidal flooding may be primarily “nuisance” severity at present, it could lead to very damaging floods by late-century based on current SLR projections (McInnes et al., 2015; Fox-Kemper et al., 2021). This finding assumes no successful future measures that ameliorates the impacts presently associated with a 2.67 m water level.

### International relevance of the results

This study focuses on eastern Australian, but our findings have global implications. For example, we find that the defining feature of locations most at-risk of the emergence of tidal flooding is having lower freeboards. This metric is known to be important globally for identifying future coastal flood hazard hotspots (e.g., Hague et al., 2023b). This means that it is likely that locations with flood thresholds close or below the heights of the highest high tides (i.e., small or negative freeboards) are likely to be at risk of transitioning to tidal flood regimes, regardless of where they are in the world. It further indicates the importance of defining, or accurately estimating flood thresholds for all coastal locations, and continuing to develop methods to do this (Hague et al., 2019; Moore and Obradovich, 2020; Mahmoudi et al., 2024). This is necessary to define the freeboard metric and hence, identify future flood hotspots in estuaries where populations may be at risk. These may be locations where undertaking detailed hydrodynamic modelling may need to be prioritised in a resource-constrained environment to best inform coastal adaptation policy.

## Conclusion

Currently, official minor, moderate and major flood thresholds are infrequently exceeded at Australian estuarine locations, for example, once a decade for moderate flood thresholds. Under less than 0.7 m SLR, we identify 13 locations that expect moderate flood impacts at least annually due to tides alone under average weather conditions. This includes Ballina in New South Wales, which is expected to see water levels that led to evacuations and widespread flooding in March 2022 occurring several days per year under 0.7 m SLR. As sea levels rise, historical riverine and compound riverine-coastal flood thresholds will be exceeded by tides alone in many Australian estuaries. Our analysis provides a lower bound for rates of present-day official flood threshold exceedances by tides, storm surges, high streamflow, and combinations of these physical drivers under SLR.

The characteristics of future compound flood hotspots differ to present-day compound flood hotspots. Locations with large riverine-driven flood peaks are currently most at risk. In contrast, locations with small tidal ranges and low flood thresholds are most at risk from increasing flood rates under SLR (e.g., Hague et al., 2023b). This suggests locations closest to mouths of estuaries are at greatest risk of the impacts of SLR. These areas are currently under-represented among locations in Australia for which official flood thresholds have been defined. This highlights the need for future work to define more flood thresholds in such locations to facilitate more comprehensive assessments of the potential for flood regime transitions. The freeboard metric used here is shown to be locally relevant but also internationally scalable, making it well suited for use in national flood hazard assessments to inform adaptation.

## Supporting information

Hague et al. supplementary material Hague et al. supplementary material

## Data Availability

Raw data were generated by various state and local government agencies and provided to the Bureau of Meteorology for the provision of the Total Flood Warning System. Lakes Entrance data are provided by Gippsland Ports, Ballina data are provided by Manly Hydraulics Laboratory and Barrack St Jetty data are provided by the Western Australia Department of Transport. Raw data owners for NSW and Queensland can be found in their respective Service Level Specification documents (Bureau of Meteorology, 2013, 2022a). Derived, quality-assured hourly water and tide levels described in sections “Quality assurance” and “Harmonic tidal analysis” are made available at http://doi.org/10.5281/zenodo.11228628. Summary metrics include tide levels and flood thresholds supporting the findings of this study as well as basic tide gauge metadata are available in Supplementary Table S1.

## Supplementary material

The supplementary material for this article can be found at http://doi.org/10.1017/cft.2024.12.
