# Supplementary material for: Eastern Australian estuaries will transition to tidal flood regimes in coming decades
Source: Camb Prism Coast Futur. 2024 Oct 4;2:e12. doi: 10.1017/cft.2024.12 (PMC12337581; doi:10.1017/cft.2024.12)
Supplement: Peer review history

# Editor report: Recommendation: Eastern Australian estuaries will transition to tidal flood regimes in coming decades — R0/PR2

**DOI:** 10.1017/cft.2024.12.pr2

**Affiliations:** College of the Environment and Ecology, Xiamen University, China

The research is intriguing as it highlights the current flooding situations and the novel approach to identifying flood regimes in Australia. It is relevant and aligns with the journal’s focus as it addresses the pressing issue of rising sea levels leading to more frequent and severe coastal flooding and its associated risks. This paper presents a well-written and straightforward analysis of the impact of sea-level rise on the freeboard of coastal areas, highlighting the need for further research and planning efforts to address the potential risks associated with coastal flooding. 

Two reviewers have provided positive feedback, suggesting minor modifications. In light of their assessments, I have recommended minor revisions for this work.

# Editor report: Decision: Eastern Australian estuaries will transition to tidal flood regimes in coming decades — R0/PR3

**DOI:** 10.1017/cft.2024.12.pr3

**Affiliations:** Geography, Cambridge University, United Kingdom of Great Britain and Northern Ireland

No accompanying comment.

# Editor report: Recommendation: Eastern Australian estuaries will transition to tidal flood regimes in coming decades — R1/PR5

**DOI:** 10.1017/cft.2024.12.pr5

**Affiliations:** College of the Environment and Ecology, Xiamen University, China

No accompanying comment.

# Editor report: Decision: Eastern Australian estuaries will transition to tidal flood regimes in coming decades — R1/PR6

**DOI:** 10.1017/cft.2024.12.pr6

**Affiliations:** Geography, Cambridge University, United Kingdom of Great Britain and Northern Ireland

No accompanying comment.
